# A Combination of L‐Triiodothyronine and Levothyroxine in the Treatment of Tyrosine Kinase Inhibitor‐Induced Hypothyroidism in Renal Cell Carcinoma: A Case Report

**DOI:** 10.1002/ccr3.70955

**Published:** 2025-11-09

**Authors:** Ya‐Juan Han, Shuo Chen, Run Miao, Zhao‐Ying Yu, Yu‐Bing Wang

**Affiliations:** ^1^ Department of Endocrinology and Metabolism Guangzhou Eighth People's Hospital, Guangzhou Medical University Guangzhou People's Republic of China; ^2^ Department of Endocrinology and Metabolism TCM‐Integrated Cancer Center of Southern Medical University Guangzhou People's Republic of China; ^3^ Department of Thoracic Surgery Guangzhou Eighth People's Hospital, Guangzhou Medical University Guangzhou People's Republic of China

**Keywords:** hypothyroidism, levothyroxine, thyroid tablet, thyroxine deiodinase dysfunction, tyrosine kinase inhibitor

## Abstract

Hypothyroidism is a common complication in advanced cancer patients taking tyrosine kinase inhibitors. For patients exhibiting suboptimal responses to standard L‐T4 therapy, timely consideration of L‐T3‐containing therapeutic regimens is crucial to prevent the progression of hypothyroidism.

**Abbreviations**
ATA: American Thyroid Association
FT3: free triiodothyronine
FT4: free tetraiodothyronine
HIF‐1: hypoxia‐inducible factor 1
L‐T3: L‐triiodothyronine
L‐T4: levothyroxine
RCC: renal cell carcinoma
rT3: reverse triiodothyronine
T3: triiodothyronine
T4: tetraiodothyronine
TG‐Ab: thyroglobulin antibody
TKI: tyrosine kinase inhibitor
TPO‐Ab: anti‐thyroid peroxidase antibody
TRAb: anti‐thyroid‐stimulating hormone antibodies
TSH: thyroid‐stimulating hormone
VEGFR: vascular endothelial growth factor receptor

## 1 Introduction

Hypothyroidism refers to a common condition in which the thyroid gland does not produce sufficient levels of thyroid hormones [1]. Although hypothyroidism is easily diagnosed and managed, it can have a detrimental effect on health and ultimately lead to death if left untreated [1]. Previous studies have demonstrated that patients treated with tyrosine kinase inhibitors (TKIs), such as sunitinib and axitinib, for advanced renal cell carcinoma (RCC) tend to have a higher incidence of thyroid dysfunction, such as hypothyroidism, thyroiditis, and thyrotoxicosis [2]. Among these thyroid dysfunctions, hypothyroidism is the most common [3].

Levothyroxine (L‐T4), an exogenous form of tetraiodothyronine (T4), has been the “gold standard” for the treatment of primary hypothyroidism for more than 60 years [4]. Despite L‐T4 monotherapy having been recommended since the 1970s, combination therapy with L‐T4 plus L‐triiodothyronine (L‐T3) has been readdressed in several clinical guidelines to accommodate special patients who remain inadequately treated and/or display persistent symptoms despite L‐T4 therapy [4]. Although the addition of synthetic L‐T3 to standard L‐T4 therapy would create a more natural treatment plan in some patients, European, UK, and American Thyroid Association (ATA) guidelines recommend this combination therapy as an individual experimental approach only in certain circumstances [4]. Since there are few reports regarding combination treatment or the corresponding changes in thyroid function or medication effects for TKI‐induced hypothyroidism in RCC patients, in the present report, we chose a case to focus on this situation.

## 2 Case History/Examination

A 65‐year‐old male patient was incidentally found to have a left kidney mass during a routine physical examination in 2004. Subsequent investigations, including a PET‐CT scan performed in January 2016 at the First Affiliated Hospital of Sun Yat‐sen University, revealed concerning findings: (1) multiple nodules and masses in both kidneys, raising suspicion of malignant tumors; (2) multiple nodules in both lungs exhibiting some metabolic activity. On January 12th, 2016, an ultrasound‐guided biopsy of the left kidney mass was conducted for the patient. The postoperative pathology report confirmed clear‐cell RCC of the left kidney. Unfortunately, no specific treatment was initiated at that time. On January 19th, 2016, the patient presented to our oncology department with complaints of “systemic metastasis of RCC over two years and worsening fatigue over the past week”. Subsequent evaluation led to a diagnosis of left renal cell carcinoma, stage IV (T4N3M1). From then until 2018, we systematically gathered in‐depth information on the clinical treatment process and its results.

**FIGURE 1:**
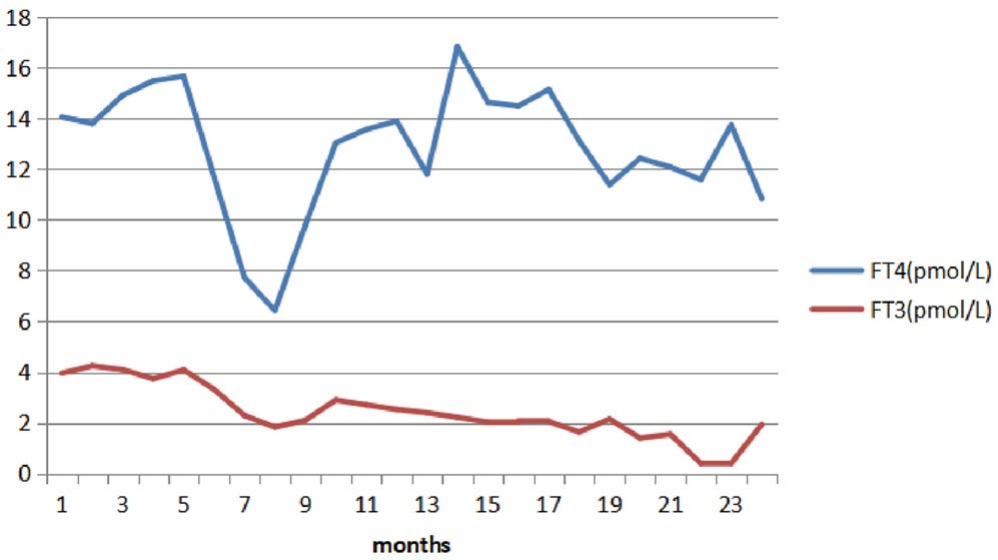
The change/trend in the patient's FT3 and FT4 levels during treatment. The FT3 and FT4 levels (*y*‐axis) were plotted against time (*x*‐axis). Different colored lines indicate different indexes of thyroid function. FT3, free triiodothyronine; FT4, free tetraiodothyronine.

**FIGURE 2:**
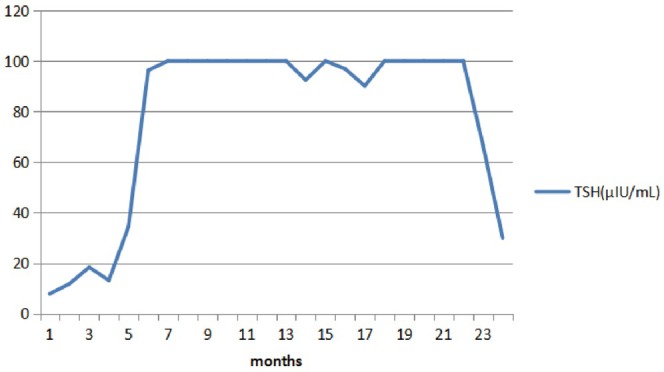
The change/trend in the patient's TSH level during treatment. The TSH level (*y*‐axis) was plotted against time (*x*‐axis). TSH, thyroid stimulating hormone.

The patient began sunitinib treatment at a dosage of 50 mg/day from the second day of hospitalization (on January 20th, 2016), without any other medications such as chemotherapy or radiotherapy. The thyroid‐stimulating hormone (TSH) level was 1.4 μIU/mL before treatment. After taking the medication, the patient experienced elevated blood pressure, peaking at 180/110 mmHg, accompanied by a fever with a maximum temperature of 39.5°C (specific time not documented in medical records). After ruling out other potential causes, the adverse reactions attributed to sunitinib were considered. As a result, the dosage of sunitinib was reduced to 37.5 mg/day on March 1st, 2016, which led to symptom relief. Subsequently, the patient continued a maintenance treatment with sunitinib at a dosage of 12.5 mg/day. On July 21st, 2016, he complained of chills, lethargy, and fatigue. Based on the hospital's protocol for thyroid function tests, free tetraiodothyronine (FT4), free triiodothyronine (FT3), and TSH were measured in the fasting state. The results showed that the FT_4_ level was 14.05 pmol/L (12–22 pmol/L), the FT3 level was 3.96 pmol/L (3.1–6.8 pmol/L), and the TSH level was 7.87 μIU/mL. Results of the anti‐thyroid peroxidase (TPO‐Ab) and thyroglobulin (TG‐Ab) antibody tests were negative. Thyroid ultrasound with color Doppler showed no obvious abnormalities. He had no previous personal or family history of thyroid disease, and there was no history of neck exposure to radiation therapy, recent fever, or viral illness. Physical examination revealed no abnormalities or significant changes in body weight. In consideration of transient mild subclinical hypothyroidism, oncologists did not administer thyroid hormone replacement therapy. However, on August 19th, 2016, thyroid function tests showed that the TSH level further increased to 11.8 μIU/mL, but the FT4 and FT3 levels remained normal. Subsequently, the patient was given oral thyroid hormone replacement therapy at 50 μg L‐T4/day. Surprisingly, on April 1st, 2017, the patient was experiencing progression of his thyroid disease, with an FT_4_ level of 6.44 pmol/L, an FT_3_ level of 1.83 pmol/L, and TSH > 100 μIU/mL, then increased the dosage of L‐T4 to 100 μg/day. On April 27th, 2017, thyroid function tests showed that the FT_4_ level was 9.78 pmol/L, the FT_3_ level was 2.1 pmol/L, and the TSH level was > 100 μIU/mL, the dosage of L‐T4 remained unchanged. On October 13th, 2017, the follow‐up ultrasound examination revealed a solid mass in the lower left limb, indicating a possible tumor metastasis. Due to potential resistance to the sunitinib treatment, the therapy was switched to oral axitinib at a dose of 5 mg/day. Meanwhile, thyroid function tests showed that the FT_4_ level was 11.8 pmol/L, the FT_3_ level was 2.4 pmol/L, and the TSH level was > 100 μIU/mL, the doctor increased the dose of L‐T4 to 150 μg/day. On December 19th, 2017, the dosage of axitinib was adjusted down to 3.75 mg/day; thyroid function tests showed that the FT_4_ level was 14.63 pmol/L, the FT_3_ level was 2.03 pmol/L, and the TSH level was > 100 μIU/mL, the dosage of L‐T4 was increased to 200 μg/day. To our disappointment, although the patient had good compliance and there was no obvious diarrhea, the same situation remained after half a year of treatment. On August 7th, 2018, thyroid function tests showed that the FT_4_ level was 13.74 pmol/L, the FT_3_ level was < 0.4 pmol/L, and the TSH level was 66.83 μIU/mL, and the patient was in a state of cachexia, accompanied by fatigue, lethargy, and poor appetite. Physical examination revealed that he had developed mild edema in both lower extremities, the heart rate fluctuated between 80 and 100 beats/min, and body temperature fluctuated between 36.5°C and 37.5°C/24 h. On August 23rd, 2018, after consulting with an endocrinologist, we switched from L‐T4 to a thyroid tablet (40 mg/day). Thyroid tablets are primarily formulated from extracts derived from porcine, bovine, and ovine thyroid glands. Biochemically, these tablets are characterized by their content of two principal thyroid hormones, T4 and T3, which are present in a 4:1 ratio. Notably, each 1 mg of the thyroid tablets contains 0.52–0.64 μg of L‐T4 and 0.13–0.15 μg of L‐T3 [5]. A week later (on August 30th, 2018), thyroid function tests showed that the FT_4_ level was 10.84 pmol/L, the FT_3_ level was 1.93 pmol/L, and the TSH level was 29.97 μIU/mL. On September 7th, 2018, according to the end‐of‐life resuscitation record, the patient was in the advanced stage of renal cancer, in a state of cachexia, with a weakened cough reflex, ultimately leading to the death from asphyxia. The change/trend in the patient's thyroid function is shown in Figures 1 and 2, and the adjustment of thyroid drug replacement therapy and sunitinib/axitinib therapy after 2017 is shown in Table 1.

**TABLE 1:** Medication adjustment during the whole treatment.

Date	FT_3_ (3.1–6.8 pmol/L)	FT_4_ (12–22 pmol/L)	TSH (0.27–4.2 μIU/mL)	Levothyroxine sodium tablets (μg/day)	Targeted treatment (mg/day)
04/01/2017	1.83 ↓	6.44 ↓	> 100 ↑↑	100	Sunitinib 12.5
04/27/2017	2.1 ↓	9.78 ↓	> 100 ↑↑	100	Sunitinib 12.5
06/07/2017	2.9↓	13.03	> 100 ↑↑	100	Sunitinib 12.5
10/13/2017	2.4 ↓	11.8 ↓	> 100 ↑↑	100	Sunitinib 12.5
10/13/2017	Due to potential resistance to the sunitinib treatment, the therapy was switched to oral axitinib (5 mg/day). Meanwhile, the dose of L‐T4 was increased to 150 μg/day				
11/20/2017	2.22 ↓	16.83	92.46 ↑↑	150	Axitinib 5
12/19/2017	2.03 ↓	14.63	> 100 ↑↑	150	Axitinib 5
12/19/2017	The dosage of axitinib was adjusted down to 3.75 mg/day. The dosage of L‐T4 was increased to 200 μg/day				
01/11/2018	2.08 ↓	14.48	96.88 ↑↑	200	Axitinib 3.75
03/08/2018	1.64 ↓	13.12	> 100 ↑↑	200	Axitinib 3.75
04/11/2018	2.15 ↓	11.38 ↓	> 100 ↑↑	200	Axitinib 3.75
05/09/2018	1.40 ↓	12.42	> 100 ↑↑	200	Axitinib 3.75
06/12/2018	1.55 ↓	12.08	> 100 ↑↑	200	Axitinib 3.75
07/11/2018	< 0.4 ↓↓	11.58 ↓	> 100 ↑↑	200	Axitinib 3.75
08/07/2018	< 0.4 ↓↓	13.74	66.83 ↑↑	200	Axitinib 3.75
08/23/2018	After consulted with endocrinologist, the treatment was changed to “Thyroid Tablets”, 40 mg per day (each 1 mg contains 0.52–0.64 μg of levothyroxine and 0.13–0.15 μg of L‐triiodothyronine)				
08/30/2018	1.93 ↓	10.84 ↓	29.97 ↑	Thyroid tablets 40 mg/day	Axitinib 3.75
09/07/2018	The patient died of asphyxia				

## 3 Differential Diagnosis

Primary hypothyroidism: The patient's TSH level was within the normal range prior to treatment. A thyroid ultrasound with color Doppler revealed no significant abnormalities. There was no prior personal or family history of thyroid disorders, and the patient had no history of neck radiation exposure, recent fever, or viral illness.

## 4 Conclusion and Results

Hypothyroidism is a common complication in advanced cancer patients taking TKIs [6]. We highly recommend thyroid hormone as replacement therapy for the treatment of TKI‐induced intractable hypothyroidism. We assume that a thyroid tablet may be a reasonable option to prevent the progression of hypothyroidism.

## 5 Discussion

Both sunitinib and axitinib are multi‐targeted TKIs commonly used as treatments for RCC [7]. TKIs are associated with a reduction in tumor proliferation, angiogenesis, and metastasis [8]. Multiple studies have reported that patients receiving TKI treatment experience a higher incidence of hypothyroidism than the general population [9, 10].

The present case of an uncommon clinical event attracted our close attention. There is no significant difference between treatments for TKI‐induced hypothyroidism and clinical hypothyroidism. Under most circumstances, TKI‐induced hypothyroidism can be improved after receiving L‐T4, with the exception of individuals who need long‐term replacement treatment. However, in this case, L‐T4 at 200 μg/day still failed to control the hypothyroidism. As far as we know, this is the first description of such an event of TKI‐induced hypothyroidism in RCC patients.

Before being treated with TKIs, the patient had normal thyroid function, and thyroid‐related autoantibodies were negative, indicating that the hypothyroidism was not autoimmune‐related but drug‐induced. We speculated that in the early stage of hypothyroidism in this patient, TKIs may have caused direct damage to the thyroid gland since the FT3 and FT4 levels were normal, but the TSH level was progressively elevated. As the patient's condition progressed, not only was the FT3/FT4 ratio gradually decreased, but the TSH level could not be inhibited by increasing the dosage of L‐T4. This phenomenon is inconsistent with common clinical events, and the possible mechanisms associated with TKI‐induced hypothyroidism (e.g., increased T4 metabolism and L‐T4 requirement) [9] could not completely explain the situation; therefore, we assumed thyroxine iodotyrosine deiodinase dysfunction.

Deiodination is an important way to regulate the biological activity of thyroid hormones. Deiodinases are enzymes that activate or inactivate thyroid hormones and can be divided into three types (D1, D2, and D3, respectively) according to tissue distribution [11]. Approximately 80% of FT3 is derived from outer‐ring deiodination of T4 activated by D1 and D2; D3 inactivates both T4 and T3 and terminates thyroid hormone action via deiodination of the inner molecular ring of T4 [12]. T4 can also be converted into biologically inactive reverse triiodothyronine (rT3) by inner‐ring deiodination [13]. These processes account for 85% of all metabolic degradation of T4 [13].

Evidence demonstrates the effect of TKIs on thyroxine iodotyrosine deiodinase and the possible mechanisms, one of which is destructive thyroiditis and/or drug‐induced thyroid atrophy [14]. Sunitinib can be directly toxic to thyroid cells, possibly by inhibiting vascular endothelial growth factor receptors (VEGFRs) [9]. Treatment with TKIs can cause destructive thyroiditis and impaired transport of T4 into cells [3]. Another proposed mechanism is the induction of D3 and inhibition of D2 [15]. This phenomenon is consistent with an animal experiment in which D3 activity was increased and D1 activity was inhibited in sunitinib‐treated mice. The potential mechanism was related to hypoxia‐inducible factor 1 (HIF‐1), which can positively regulate the gene encoding D3 [16]. Although HIF‐1 cannot be directly activated by TKIs, the anti‐angiogenic effects of TKIs can indirectly upregulate the expression of HIF‐1 [17].

Therefore, we assume that the patient's hypothyroidism may be induced by TKI‐associated thyroxine iodotyrosine deiodinase dysfunction (unable to detect rT3 due to limited conditions). Since treatment with a dosage of L‐T4 (200 μg/day) was unsatisfactory, we switched to a thyroid tablet combination of both L‐T4 and L‐T3 [5]. After a week of treatment, the patient's thyroid function was significantly improved, which further confirmed our hypothesis. However, it is necessary to accumulate similar cases for verification and more in‐depth mechanism research in the future. This may include examining the deiodinase gene expression, measuring rT3 levels, and elucidating the conversion of T4 to T3.

In summary, although L‐T4 replacement remains the established treatment for TKI‐induced hypothyroidism and demonstrates efficacy in most instances, it failed to alleviate the patient's symptoms in this particular case. Particularly concerning is the one‐year delay in implementing the effective therapeutic adjustment of substituting L‐T4 with thyroid tablets. This case highlights the necessity for endocrinologists and oncologists to re‐evaluate conventional treatment protocols. For patients exhibiting suboptimal responses to standard L‐T4 therapy, timely consideration of L‐T3‐containing therapeutic regimens is crucial to prevent the progression of hypothyroidism.

## Author Contributions

**Ya‐Juan Han:** writing – original draft. **Shuo Chen:** writing – original draft. **Run Miao:** writing – review and editing. **Zhao‐Ying Yu:** writing – review and editing. **Yu‐Bing Wang:** conceptualization.

## Ethics Statement

This case report aligns with the principles of the Declaration of Helsinki and national guidelines. It involves a retrospective analysis of existing data and has received an exemption from ethics approval by the Guangzhou Eighth People's Hospital.

## Consent

Written informed consent was obtained from the patient prior to study enrollment.

## Conflicts of Interest

The authors declare no conflicts of interest.

## Data Availability

The data that support the findings of this study are available on request from the corresponding author. The data are not publicly available due to privacy or ethical restrictions.
